# Impact of Interferon-Based Therapy on Hepatitis C-Associated Rheumatic Diseases: A Nationwide Population-Based Cohort Study

**DOI:** 10.3390/jcm10040817

**Published:** 2021-02-17

**Authors:** Jur-Shan Cheng, Yu-Sheng Lin, Jing-Hong Hu, Ming-Yu Chang, Hsin-Ping Ku, Rong-Nan Chien, Ming-Ling Chang

**Affiliations:** 1 Clinical Informatics and Medical Statistics Research Center, College of Medicine, Chang Gung University, Taoyuan 333323, Taiwan; jscheng@mail.cgu.edu.tw; 2 Department of Emergency Medicine, Chang Gung Memorial Hospital, Keelung 20401, Taiwan; find94132@yahoo.com; 3 Department of Cardiology, Chang Gung Memorial Hospital, Taoyuan 333423, Taiwan; ma3958@gmail.com; 4 Healthcare Center, Chang Gung Memorial Hospital, Taoyuan 333423, Taiwan; 5 Department of Internal Medicine, Chang Gung Memorial Hospital, Yunlin 63862, Taiwan; a3237184@gmail.co; 6 Division of Pediatric Neurologic Medicine, Chang Gung Children’s Hospital, Taoyuan 333423, Taiwan; p123073@gmail.com; 7 Division of Pediatrics, Chang Gung Memorial Hospital, Keelung 20401, Taiwan; 8 Department of Medicine, College of Medicine, Chang Gung University, Taoyuan 333423, Taiwan; ronald@cgmh.org.tw; 9 Division of Gastroenterology, Department of Gastroenterology and Hepatology, Chang Gung Memorial Hospital, Taoyuan 333423, Taiwan

**Keywords:** HCV, rheumatic, interferon, mortality

## Abstract

Whether hepatitis C virus (HCV) infection-associated risk of rheumatic diseases is reversed by anti-HCV therapy remain elusive. A nationwide population-based cohort study of the Taiwan National Health Insurance Research Database was conducted. Of 19,298,735 subjects, 3 cohorts (1:4:4, propensity score-matched), including HCV-treated (6919 HCV-infected subjects with interferon and ribavirin therapy ≥ 6 months), HCV-untreated (*n* = 27,676) and HCV-uninfected (*n* = 27,676) cohorts, were enrolled and followed (2003–2015). The HCV-uninfected cohort had the lowest cumulative incidence of rheumatic diseases (95% confidence interval (CI): 8.416–10.734%), while HCV-treated (12.417–17.704%) and HCV-untreated (13.585–16.479%) cohorts showed no difference in the cumulative incidences. Multivariate analyses showed that HCV infection (95% CI hazard ratio (HR): 1.54–1.765), female sex (1.57–1.789), age ≥ 49 years (1.091–1.257), Charlson comorbidity index ≥ 1 (1.075–1.245), liver cirrhosis (0.655–0.916), chronic obstruction pulmonary disease (1.130–1.360), end-stage renal disease (0.553–0.98), diabetes mellitus (0.834–0.991) and dyslipidemia (1.102–1.304) were associated with incident rheumatic diseases. Among the 3 cohorts, the untreated cohort had the highest cumulative incidence of overall mortality, while the treated and un-infected cohorts had indifferent mortalities. Conclusions: HCV infection, baseline demographics and comorbidities were associated with rheumatic diseases. Although HCV-associated risk of rheumatic diseases might not be reversed by interferon-based therapy, which reduced the overall mortality in HCV-infected patients.

## 1. Introduction

Hepatitis C virus (HCV) is a human pathogen responsible for acute and chronic liver disease that infects an estimated 150 million individuals worldwide [1]. In addition to hepatic complications including cirrhosis and hepatocellular carcinoma, HCV may cause many extrahepatic complications such as diabetes mellitus (DM), hypolipidemia, cardiovascular events [1], and rheumatic diseases [2]. HCV is both hepatotropic and lymphotropic [3]. HCV lymphotropism represents the most important step in the pathogenesis of virus-related immunological diseases [4], especially rheumatic diseases. Rheumatologic extrahepatic manifestations are observed in 2% to 38% of HCV-infected patients [5], and this variability is attributed to the various geographic region and design of the studies [6,7,8]. Moreover, HCV antibodies were found in 18.5% among patients admitted to the rheumatology ward [9], being higher than the estimated global prevalence (2.2–2.8%) of HCV infection [10]. The Hispanoamerican Study Group of Autoimmune Manifestations associated with Hepatitis C Virus (HISPAMEC) Registry showed that the systemic autoimmune diseases most associated with chronic HCV infection were Sjogren syndrome (SS), rheumatoid arthritis (RA) and systemic lupus erythematosus (SLE) [11]. Specifically, the co-prevalence of HCV and SS ranged from 49% [12] to 80% [13], HCV infection was found in 13% of a large series of Spanish patients with SS [14], and sicca symptoms were reported in 11% of French HCV patients [15]. HCV infection was also associated with increased RA risks [16,17], and the pooled prevalence of RA was 4.5% (0.6–25.7%) of chronic HCV-infected patients in East Asia [2]. Moreover, the prevalence of HCV infection among SLE patients was found to be 10% [18].

The combination of pegylated interferon (Peg-IFN) and ribavirin has provided a “cure” for a considerable proportion of patients with chronic hepatitis C infection (CHC), particularly in patients with a favorable interferon λ 3 (IFNL3) genotype [1]. These cure rates were further improved by replacing interferon-based therapy with potent, direct-acting antiviral agents (DAAs) [1], and the sustained virological response rate (SVR) to DAA in HCV-infected patients is approaching 100% [19]. However, some HCV-associated complications such as cardiometabolic and oncogenic events cannot be reversed, even after viral clearance [1,20,21]. Whether the HCV-associated risk of rheumatic diseases can be attenuated after the completion of anti-HCV therapy thus is still a crucial issue of public health in the era of DAA to eradicate HCV infection but remains elusive.

Accordingly, we conducted a nationwide population-based cohort study in Taiwan, where HCV infection is rampant [22]. The impacts of HCV infection and anti-HCV therapy on the risk of rheumatic diseases were investigated by comparing the cumulative incidences of rheumatic diseases and of the overall mortalities among HCV-infected subjects with and without anti-HCV therapy and the subjects without HCV infection, based on data from the Taiwan National Health Insurance Research Database (TNHIRD). This database provides medical information of the nationwide population, which comprises 26,573,661 individuals.

## 2. Methods

### 2.1. TNHIRD Samples and Measurements

This population-based retrospective cohort study used nation-level data, including the National Health Insurance (NHI) administrative database, the Cancer Registry Database, and the Death Registry Database of Taiwan. The mandatory, single-payer NHI program provides comprehensive coverage including ambulatory care, hospital services, laboratory tests, and prescription drugs. Over 99% of the population is enrolled in the program and approximately 90% of the healthcare organizations are contracted with NHI Administration. Given that Taiwan is a hyperendemic area for hepatitis B virus (HBV) infection, which is highly oncogenic, causes many hepatic complications and prominently biases the phenotype of HCV infection [23], the subjects diagnosed with HBV infection in the observation period (2003–2015), or with any baseline rheumatic diseases including RA (International Classification of Disease, Ninth. Revision, Clinical Modification (ICD-9-CM) code (714)), ankylosing spondylitis (ICD-9-CM code (720)) [24], psoriatic arthopathy (ICD-9-CM code (696.0)), sicca syndrome (also called SS) (ICD-9-CM code (710.2)), systemic sclerosis (ICD-9-CM code (710.1)), SLE (ICD-9-CM code (710.0)), Behcet’s syndrome (ICD-9-CM code (136.1)) [25], Raynaud’s syndrome (ICD-9-CM code (443.0)), polyarteritis nodosa and allied conditions (ICD-9-CM code (446)) [26], and psoriasis (ICD-9-CM code (696.X)) [27] or mortality occurred prior to 6 months after completing anti-HCV treatment (the baseline), when it is the time to ensure therapeutic response, were excluded.

The HCV-treated cohort included subjects who had a HCV RNA test and received ribavirin and pegylated interferon (Peg-IFN) in 2003–2015. Their first HCV test was assumed to be the index date of diagnosis. The baseline for the HCV-treated cohort was the date of 6 months after completing the combination therapy. Untreated HCV-infected patients were those who had been examined for HCV infection (HCV antibody or HCV RNA test) (their first HCV test was the index date), were diagnosed with HCV (The International Classification of Diseases, Ninth Revision, Clinical Modification (ICD-9-CM) codes: 070.41, 070.44, 070.51, 070.54, 070.70, 070.71, V02.62), were prescribed hepatoprotective agents (silymarin, liver hydrolysate, choline bitartrate, or ursodeoxycholic acid), but did not receive any anti-HCV therapy (ribavirin or peg-interferon). HCV-uninfected individuals were those who did not have a HCV diagnosis (ICD-9-CM) or tests for HCV infection, and received no hepatoprotective agents or anti-HCV therapy, and they were classified as being HCV-uninfected. The HCV-treated cohort was matched with untreated HCV-infected patients (HCV-untreated cohort) and with HCV-uninfected individuals (HCV-uninfected cohort) through a propensity score-matching method indicating the probability of receiving the combination therapy, estimated by using a logistic model. The covariates in the model included sex (male, female), age (20–39, 40–49, 50–59, ≥60), NHI registration location (city, township, rural area), Charlson Comorbidity Index (CCI) score (0, 1, ≥2), and year of the index date (2003–2006, 2007–2009, 2010–2012). This method was used to assure that the HCV-treated cohort and the selected counterparts were comparable in observed characteristics. The baselines for the HCV-untreated and HCV-uninfected cohorts were assigned according to the period from the index date to the baseline of their matched counterparts of the HCV-treated cohort, and subjects with rheumatic disease or mortality occurred before the baselines were not selected. The index date of the HCV-uninfected individuals was the date of one of their physician visits randomly selected from their claims database. The matching process for the 3 cohorts is shown in Supplementary Figure S1.

Outcomes were defined as the development of rheumatic diseases as mentioned above. Subjects were followed until the date of the event, death, or the end of follow-up (31 December 2015), whichever came first. Dates of death were adopted from the Death Registry database. For the HCV-treated group, only the rheumatic disease or mortality occurred 6 months after the complement of anti-HCV therapy (the baseline) were recorded.

### 2.2. Statistical Analysis

All statistical analyses were performed using the Statistical Analysis System (SAS version 9.4, SAS Institute Inc., Cary, NC, USA) software. Continuous variables were analyzed using a Student’s *t*-test or analysis of variance, as appropriate, and categorical variables were analyzed using a Chi-square test or Fisher’s exact test, as appropriate. Cumulative incidences of outcomes were estimated and compared by using the modified Kaplan–Meier method and the Gray method, with death being a competing risk event. Sub-distribution hazards models for competing risks, an extension of Cox proportional hazards models taking competing mortality into consideration, were used to estimate adjusted hazard ratio of developing rheumatic diseases, adjusting for age, sex, NHI registration location, the CCI score, year of the index date, and comorbid liver cirrhosis, chronic obstructive pulmonary disease (COPD), end-stage renal disease (ESRD), DM, hypertension, dyslipidemia, cardiovascular events (including percutaneous coronary intervention, coronary artery bypass graft, myocardial infarction, heart failure, cardiogenic shock, and peripheral vascular disease), stroke, nonalcoholic fatty liver disease (NAFLD), alcoholic liver disease (ALD), and autoimmune liver disease. Statistical significance was defined at the 5% level.

### 2.3. Ethics Approval and Consent to Participate

The study protocol conformed to the ethical guidelines of the 1975 Declaration of Helsinki and was approved by the local Institutional Review Board. The need for consent was waived because the national-level data used in this study were de-identified by encrypting personal identification information.

## 3. Results

### 3.1. Baseline Characteristics

**Figure 1:**
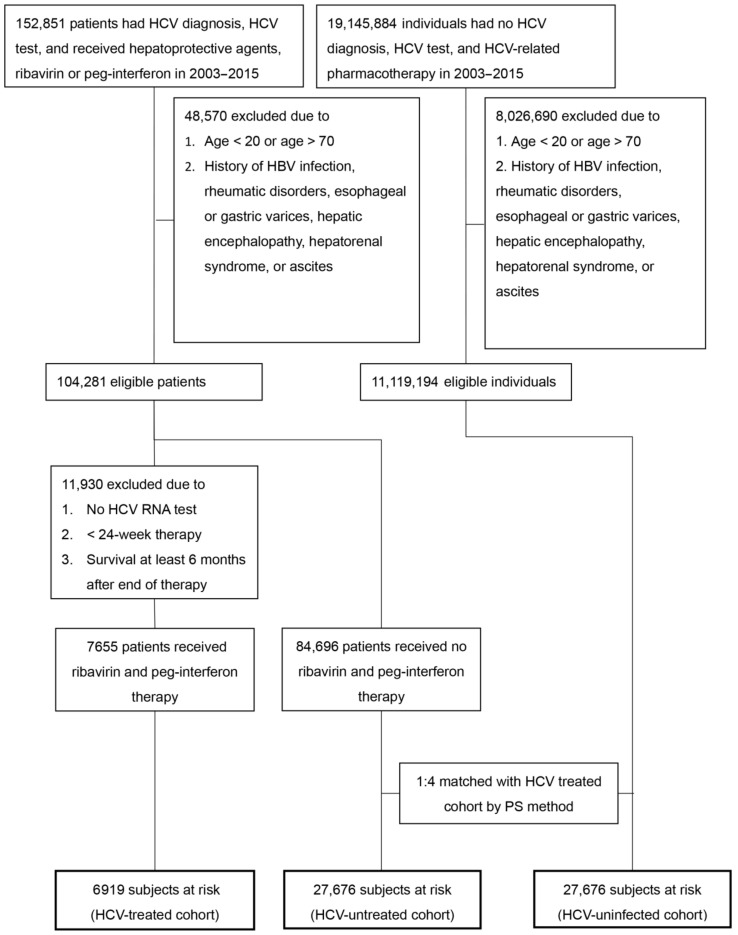
Flow chart of TNHIRD study subjects selection. TNHIRD: Taiwan National Health Insurance Research Database; HCV: hepatitis C virus; Peg-IFN: pegylated interferon; PS: propensity score.

From a total of 19,298,735 individuals between 1 January 2003 and 31 December 2015, 11,223,475 patients without HBV infection and baseline rheumatic diseases were identified; 104,281 patients with HCV infection and 11,119,194 patients without HCV infection were eligible for the study. Of all, 3 cohorts including HCV-treated (*n* = 6919), HCV-untreated (*n* = 27,676) and HCV-uninfected (*n* = 27,676) cohorts were enrolled (Figure 1). The 3 cohorts were matched with the propensity scores (1:4:4), did not differ in demographic factors, residency, CCI score and index year, which were the covariates in the models to calculate propensity scores, although baseline comorbidities were not similar (Table 1). Compared with HCV-untreated cohorts, the HCV-treated cohort had higher rates of baseline cirrhosis, comparable rates of COPD, but lower rates of other comorbidities. Compared with the HCV-uninfected cohort, the HCV-treated cohort had higher rates of most comorbidities including cirrhosis, comparable rates of DM and cardiovascular events, but lower rates of dyslipidemia and stroke. Compared with the HCV-uninfected cohort, the HCV-untreated cohort had higher rates of all baseline comorbidities except stroke. To lineate the HCV-associated complications, we compared the baseline factors between the HCV-infected cohort, which was a combination of the HCV-treated and HCV-untreated cohorts, and HCV-uninfected cohort. The HCV-infected cohort had higher rates of all baseline comorbidities except indifferent rates of dyslipidemia and lower rates of stroke than the HCV-uninfected cohort (Supplementary Figure S2).

### 3.2. Cumulative Incidences and Associated Factors of Rheumatic Diseases

**Figure 2:**
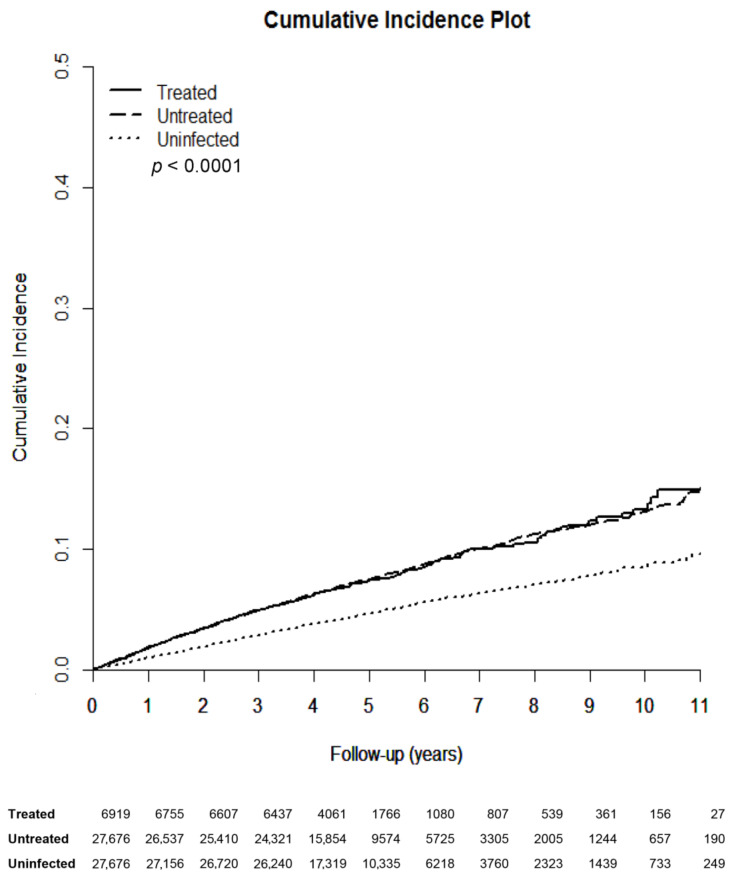
Cumulative incidences of rheumatic diseases among the 3 TNHIRD cohorts including HCV-treated, HCV-untreated and HCV-uninfected cohorts. TNHIRD: Taiwan National Health Insurance Research Database; HCV: hepatitis C virus.

**Figure 3:**
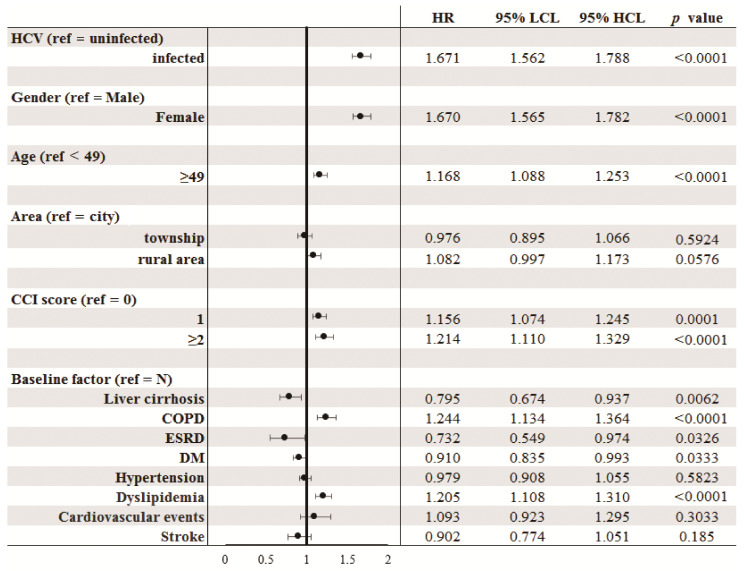
Forrest plot of factors associated with incident rheumatic diseases in the 2 TNHIRD cohorts: HCV-positive (untreated) and HCV-negative (combination of treated and uninfected) cohorts. TNHIRD: Taiwan National Health Insurance Research Database; HR: hazards ratio; LCL: lower confidence interval limit; HCL: higher confidence interval limit; HCV: hepatitis C virus; CCI: Charlson Comorbidity Index score; COPD: Chronic obstructive pulmonary disease; ESRD: end-stage renal disease; DM: diabetes mellitus; NAFLD: Nonalcoholic fatty liver disease; ALD: alcoholic liver disease.

The HCV-treated, -untreated, and -uninfected cohorts were followed up until 2015 or death, with the longest observation of 11 years. Rheumatic diseases occurred cumulatively at 11 years in 14.95%, 14.999%, and 9.535% of the HCV-treated, -untreated, and -uninfected cohorts, respectively (Figure 2, Table 2). The HCV-uninfected cohort had the lowest cumulative incidence of rheumatic diseases among the 3 cohorts. However, no difference of cumulative incidences of rheumatic diseases was identified between the HCV-treated and HCV-untreated cohorts. The multivariate analysis of the 3 cohorts showed, compared with the HCV-uninfected cohort, that both the HCV-treated and HCV-untreated cohorts had higher hazard ratios (HRs) to develop rheumatic disease. In addition, female sex, baseline age ≥ 49 years, CCI score ≥ 1, baseline COPD and dyslipidemia were associated with increased HRs of rheumatic diseases, while baseline liver cirrhosis, ESRD and DM were associated with decreased HRs of rheumatic diseases (Supplementary Figure S2). Given that HCV-treated and HCV-untreated cohorts yielded similar HRs to develop rheumatic diseases, we thus combined HCV-treated and HCV-untreated cohorts to form the HCV-infected cohort as mentioned above and compared the HCV-infected cohort with the HCV-uninfected cohort to view the impact of HCV infection on the development of rheumatic diseases. In addition to sex, age, CCI score, baseline COPD, dyslipidemia, cirrhosis, ESRD and DM, HCV infection was significantly associated with the development of rheumatic diseases, with a HR of 1.649 (Figure 3).

### 3.3. Cumulative Incidences of Mortality.

Of the 3 cohorts, the HCV-untreated cohort had the highest cumulative incidence (29.163%) of overall mortality at 11 years (*p* < 0.0001). The HCV-treated and HCV-uninfected cohorts yielded indifferent mortality rates (*p* = 0.1796) (Table 3).

## 4. Discussion

The most compelling results of the current study are as follows: (1) The HCV-uninfected cohort had the lowest cumulative incidence of rheumatic diseases among the 3 cohorts, while indifferent cumulative incidences were identified between the HCV-treated and HCV-untreated cohorts. (2) HCV infection, female gender, baseline age ≥ 49 years, CCI score ≥ 1, baseline COPD and dyslipidemia were associated with increased HRs of rheumatic diseases, while baseline liver cirrhosis, ESRD and DM were associated with decreased HRs. (3) The HCV-untreated cohort had the highest cumulative incidence of overall mortality at 11 years, while HCV-treated and HCV-uninfected cohorts yielded indifferent mortality rates.

The higher rate of baseline cirrhosis in the HCV-treated than the HCV-untreated cohorts of TNHIRD was coincided with the fact that only patients with significant fibrosis were reimbursed with anti-HCV therapy [28], and the other different baseline variables between these 2 cohorts highlight the idea that patients with comorbidities were ineligible for the interferon-based therapy and had been excluded for anti-HCV therapy. The different rates in baseline variables between HCV-infected and HCV-uninfected cohorts were consistent with the phenomenon that HCV infection elicits many cardiometabolic events and hypolipidemia [1]. Therefore, the baseline comparisons of the 3 cohorts supported the reliability of the data based on TNHIRD.

The fact that the HCV-uninfected cohort had the lowest cumulative incidence of rheumatic diseases, and HCV infection increased the HR of developing rheumatic diseases based on multivariate analyses, endorsed the concept that HCV infection might cause rheumatic diseases, despite the fact that some studies did not support the participation of HCV infection in the pathogenesis of RA [29,30,31]. However, given that the HRs in developing rheumatic diseases between the HCV-treated and HCV-untreated cohorts were indifferent, the HCV-associated risk of rheumatic diseases might not be attenuated by interferon-based anti-HCV therapy. In particular, cryoglobulinemic vasculitis represents the prototype of HCV-related rheumatic diseases [3]; long-term mixed cryoglobulinemia after SVR is common since cryoglobulin-generating B lymphocytes might have reached an HCV-independent autonomous phase before viral clearance [32]. HCV-associated rheumatic disease therefore might persist despite viral clearance. Moreover, whether interferon-based therapy reduces the risk of RA had remained conflicting [33,34], and interferon-based anti-HCV therapy may work as a “trigger” for RA [35,36] or SLE [37] had been shown in some case reports. Although treatment with interferon-alpha may lead to substantial clinical improvement of HCV-related arthritis even without a complete biochemical or virological response [34], autoimmune diseases indeed occur in 4% to 19% of patients receiving interferon-based anti-HCV therapy and the associated symptoms developed between 2 weeks and 7 years after initiation of therapy [38].The interferon-based anti-HCV therapy thus has been contraindicated for many rheumatologic autoimmune/inflammatory diseases based on the concern of triggering rheumatic diseases. New oral interferon-free combinations of various DAAs offer an opportunity for HCV-infected patients with rheumatic diseases to be cured with a short treatment duration and a low risk of side effects [39]. However, SVR following DAA might lead to immune reconstitution as tuberculosis reactivation had been reported [40]. Whether DAA therapy precisely attenuates the risks of HCV-associated rheumatic disease without introducing other harm as mentioned above [40] demands further investigation.

On the other hand, that female sex and baseline age ≥ 49 years are positively associated with the increased HRs of rheumatic diseases is consistent with the fact that female sex and old age had been identified as risk factors for RA [41]. CCI score ≥ 1 and baseline COPD were associated with increased HRs of rheumatic diseases, which coincides with the fact that comorbidities including respiratory disease were more common in patients with RA at diagnosis than controls [42]. Patients with rheumatic diseases have increased prevalence of metabolic syndrome including dyslipidemia [43], and acute myocardial infarction risk increased by 38% [44] in RA patients might explain why dyslipidemia were associated with increased HR of rheumatic diseases. Of note, the fact that baseline liver cirrhosis, ESRD and DM are associated with reduced HRs of rheumatic diseases is a novel finding. Interestingly, the connections with cirrhosis are variable among different rheumatic diseases. For example, the overall incidences of cirrhosis were reported to be lower in the RA cohort than in the non-RA cohort [45,46], while patients with psoriasis were found to have increased risk of cirrhosis over patients without psoriasis [46].With regard to ESRD and DM, in contrast to their negative associations with the rheumatic disease risks, chronic kidney disease is a common complication of rheumatic diseases [47]; patients undergoing hemodialysis therapy may develop serious rheumatic complications [48], newly diagnosed RA patients are at higher risk of DM [49] and the prevalence of DM is higher in patients with psoriatic arthritis compared with the general population [50]. That rheumatic diseases might be mistaken as ESRD- or DM-related complications in patients with ESRD and DM potentially explains the aforementioned paradox.

Among the 3 cohorts, the HCV-untreated cohort yielded the highest overall mortality, which might be caused by other HCV-associated events such as cirrhosis, HCC or cardiometabolic events [1] other than rheumatic disease-associated complications, since HCV-treated and HCV-uninfected cohorts had indifferent mortalities, although the latter obviously had a lower risk of rheumatic diseases. This phenomenon indicates the importance to prescribe anti-HCV therapy in HCV-infected patients in decreasing overall mortality, regardless of the risk for rheumatic diseases.

There are limitations recognized in the current study. First, because linking the results from TNHIRD to the laboratory results of individual patients was forbidden for privacy protection, the correlation of SVR with rheumatic diseases could not be identified. However, we are confident in the antiviral efficacy in the HCV-treated cohort since interferon-based therapy for HCV infection generally achieves an SVR rate up to 90% in Taiwan [51], where a favorable genetic variation in IFNL3 is prevalent [51]. Second, as mentioned above, interferon-based therapy might elicit rheumatic diseases in SVR patients [35,36,37,38] and blunt the impact of viral clearance in attenuating rheumatic disease risks. Third, because most of the rheumatic diseases accounted for the minority of the whole population and our preliminary statistical tests did not show any significance for any individual rheumatic disease, we thus had put all rheumatic disorders together as rheumatic diseases to yield the maximal statistical power. Some specific rheumatic disorders might have different connections with HCV infection or anti-HCV therapy. Anyhow, that SVR did not reduce the incidences of SLE and RA in CHC patients [52] supported our observation. Future prospective studies in other independent large cohorts with identifiable SVR following DAA therapy, subgroup analyses for specific rheumatic disorders and sophisticated molecular investigations are required to elucidate the fundamental mechanisms underlying the findings described here.

Taken together, HCV infection, female sex, baseline age ≥ 49 years, and other comorbidities were associated with risks of rheumatic diseases. Although interferon-based therapy did not attenuate the rheumatic disease risk, it indeed decreased the overall mortality of HCV-infected patients. These findings may merit further study for preventing or treating rheumatic diseases in HCV-infected patients.

## Figures and Tables

**Table 1:** Baseline characteristics of the 3 HCV cohorts of TNHIRD.

	(1)	(2)	(3)	*p* Values		
	Treated	Untreated	Uninfected	(1)–(2)	(1)–(3)	(2)–(3)
***n***	6919	27,676	27,676			
**Gender, *n*, (%)**						
**Male**	3832, (55.38)	15,328, (55.38)	15,328, (55.38)	1	1	1
**Female**	3087, (44.62)	12,348, (44.62)	12,348, (44.62)			
**Age range (years), *n*, (%)**						
**20–39**	1312, (18.96)	5247, (18.96)	5248, (18.96)	1	1	1
**40–49**	1811, (26.17)	7243, (26.17)	7244, (26.17)			
**50–59**	2443, (35.31)	9774, (35.32)	9772, (35.31)			
**≥60**	1353, (19.55)	5412, (19.55)	5412, (19.55)			
**Area, *n*, (%)**						
**city**	1482, (21.42)	5928, (21.42)	5928, (21.42)	1	1	1
**township**	2174, (31.42)	8696, (31.42)	8696, (31.42)			
**rural area**	3263, (47.16)	13,052, (47.16)	13,052, (47.16)			
**CCI score, *n*, (%)**						
**0**	3443, (49.76)	13,774, (49.77)	13,772, (49.76)	0.9999	1	0.9998
**1**	2138, (30.90)	8550, (30.89)	8552, (30.90)			
**≥2**	1338, (19.34)	5352, (19.34)	5352, (19.34)			
**index_year, *n*, (%)**						
**2003–2006**	3601, (52.05)	14,404, (52.05)	14,404, (52.05)	0.9997	1	0.9992
**2007–2009**	2274, (32.87)	9099, (32.88)	9096, (32.87)			
**2010–2012**	1044, (15.09)	4173, (15.08)	4176, (15.09)			
**Baseline factor, *n*, (%)**						
**Liver cirrhosis**	695, (10.04)	1685, (6.09)	9, (0.03)	<0.0001	<0.0001	<0.0001
**COPD**	775, (11.2)	3160, (11.42)	2548, (9.21)	0.6114	<0.0001	<0.0001
**ESRD**	47, (0.68)	722, (2.61)	81, (0.29)	<0.0001	<0.0001	<0.0001
**DM**	1320, (19.08)	6166, (22.28)	5004, (18.08)	<0.0001	0.0549	<0.0001
**Hypertension**	2011, (29.06)	9485, (34.27)	7422, (26.82)	<0.0001	0.0002	<0.0001
**Dyslipidemia**	815, (11.78)	5268, (19.03)	4815, (17.4)	<0.0001	<0.0001	<0.0001
**Cardiovascular events**	165, (2.38)	1059, (3.83)	685, (2.48)	<0.0001	0.6642	<0.0001
**Stroke**	227, (3.28)	1369, (4.95)	1407, (5.08)	<0.0001	<0.0001	0.4593
**NAFLD**	724 (10.46)	2425 (8.76)	188 (0.68)	<0.0001	<0.0001	<0.0001
**ALD**	105 (1.52)	653 (2.36)	20 (0.07)	<0.0001	<0.0001	<0.0001
**Autoimmune liver disease**	0	0	0			

TNHIRD: Taiwan National Health Insurance Research Database; HCV: hepatitis C virus; CCI: Charlson Comorbidity Index; COPD: Chronic obstructive pulmonary disease; ESRD: end-stage renal disease; DM: diabetes mellitus; NAFLD: nonalcoholic fatty liver disease; ALD: alcoholic liver disease.

**Table 2:** Comparison of the cumulative incidences of rheumatic diseases among (1) HCV-treated, (2) HCV-untreated and (3) HCV-uninfected cohorts.

Rheumatic Disorders	(1) Treated	(2) Untreated	(3) Uninfected	*p* Values			
				(1)(2)(3)	(1)–(2)	(1)–(3)	(2)–(3)
Number	6919	27,676	27,676				
Follow-up (years), mean ± SD	4.61 ± 1.90	4.62 ± 1.07	4.89 ± 1.96				
Event number, *n* (%)	503 (7.27)	2140 (7.73)	1310 (4.73)				
Competing mortality, *n* (%)	281 (4.06)	3478 (12.57)	1316 (4.10)				
Cumulative incidence, % (95% CI)	14.95 (12.417–17.704)	14.999 (13.585–16.479)	9.535 (8.416–10.734)	<0.0001	0.8316	<0.0001	<0.0001

CI: confidence interval.

**Table 3:** Comparison of the cumulative incidences of overall mortality among(1) HCV-treated, (2) HCV-untreated and (3) HCV-uninfected cohorts.

Overall Mortality	(1) Treated	(2) Untreated	(3) Uninfected	*p* Values			
				(1)(2)(3)	(1)–(2)	(1)–(3)	(2)–(3)
Number	6919	27,676	27,676				
Follow-up (years), mean ± SD	4.82 ± 1.84	4.86 ± 2.03	5.03 ± 1.91				
Event number, *n* (%)	304 (4.39)	3669 (13.26)	1170 (4.23)				
Cumulative incidence, % (95% CI)	13.662 (11.389–16.140)	29.163 (27.218–31.133)	9.99 (8.548–11.559)	<0.0001	<0.0001	0.1796	<0.0001

CI: confidence interval.

## Supplementary Materials

The following are available online at https://www.mdpi.com/2077-0383/10/4/817/s1, Figure S1: Steps of the matching process, Figure S2: Comparison of the baseline factors between the HCV-infected cohort (HCV-treated and HCV-untreated cohorts), and HCV-uninfected cohort.
