# Supplementary figures and images for: Impact of Interferon-Based Therapy on Hepatitis C-Associated Rheumatic Diseases: A Nationwide Population-Based Cohort Study
Source: J Clin Med. 2021 Feb 17;10(4):817. doi: 10.3390/jcm10040817 (PMC7922671; doi:10.3390/jcm10040817)

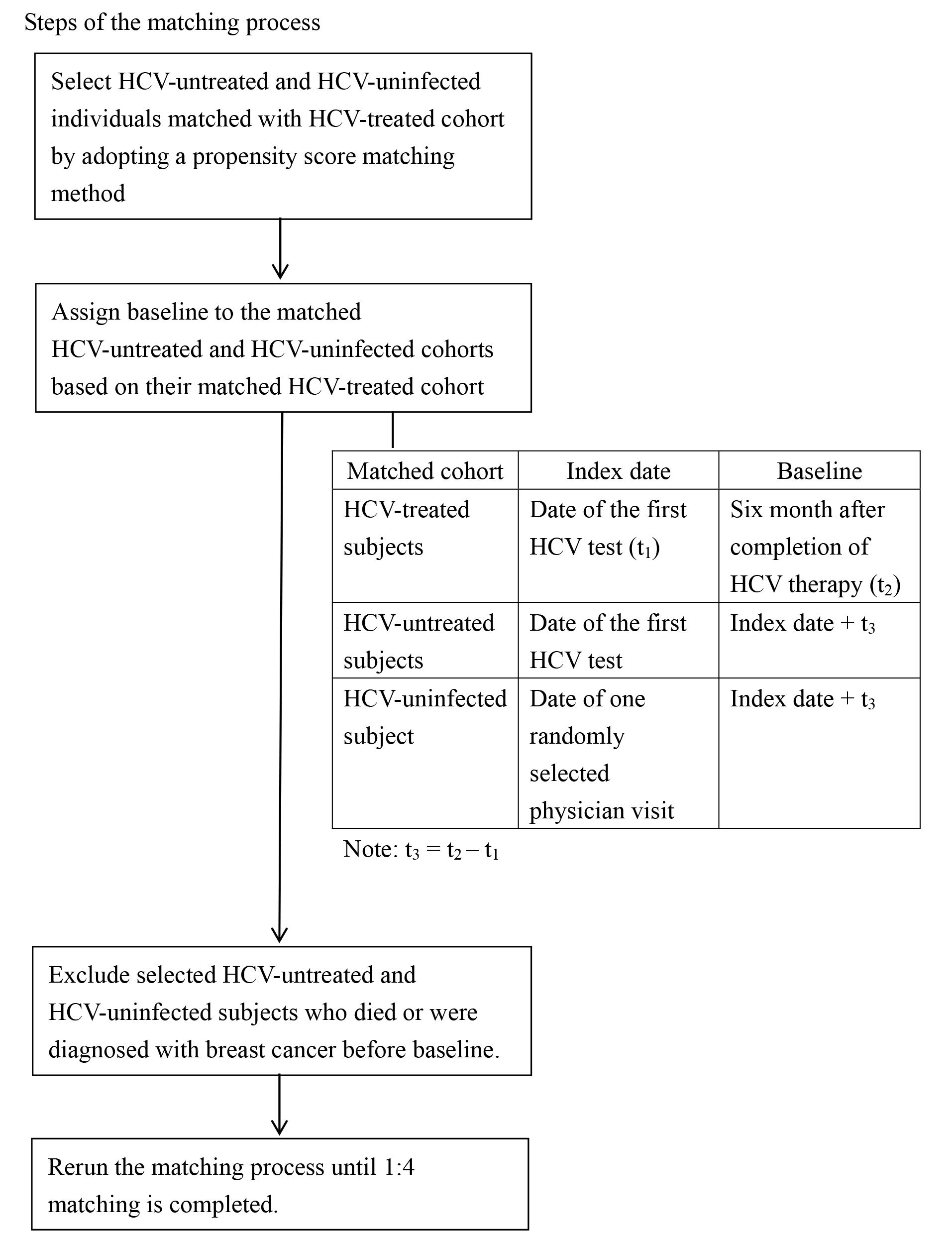

Supplement: Supplementary file 1 [file jcm-10-00817-s001.zip › jcm-1091400-supplementary/jcm-1091400-suppl/Supplementary Figure S1.tif]

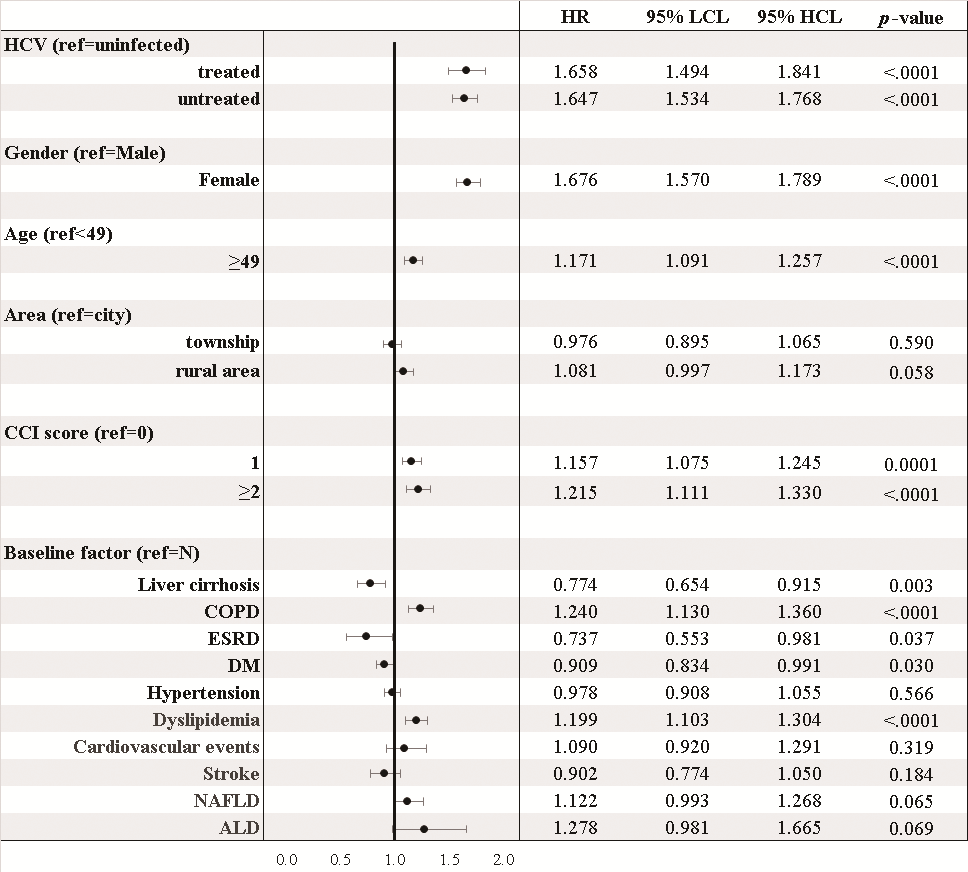

Supplement: Supplementary file 1 [file jcm-10-00817-s001.zip › jcm-1091400-supplementary/jcm-1091400-suppl/Supplementary Figure S2.tif]
